# Transglutaminase 2-mediated histone monoaminylation and its role in cancer

**DOI:** 10.1042/BSR20240493

**Published:** 2024-08-23

**Authors:** Huapeng Li, Jinghua Wu, Nan Zhang, Qingfei Zheng

**Affiliations:** 1 Molecular, Cellular, and Developmental Biology Graduate Program, The Ohio State University, Columbus, OH 43210, U.S.A.; 2 Department of Radiation Oncology, College of Medicine, The Ohio State University, Columbus, OH 43210, U.S.A.; 3 Center for Cancer Metabolism, James Comprehensive Cancer Center, The Ohio State University, Columbus, OH 43210, U.S.A.; 4 Department of Biological Chemistry and Pharmacology, College of Medicine, The Ohio State University, Columbus, OH 43210, U.S.A.; 5 Department of Medicinal Chemistry and Molecular Pharmacology, College of Pharmacy, Purdue University, IN, U.S.A.

**Keywords:** cancer biology, epigenetics, gene transcription, histone monoaminylation, transglutaminase 2 (TGM2)

## Abstract

Transglutaminase 2 (TGM2) has been known as a well-characterized factor regulating the progression of multiple types of cancer, due to its multifunctional activities and the ubiquitous signaling pathways it is involved in. As a member of the transglutaminase family, TGM2 catalyzes protein post-translational modifications (PTMs), including monoaminylation, amide hydrolysis, cross-linking, etc., through the transamidation of variant glutamine-containing protein substrates. Recent discoveries revealed histone as an important category of TGM2 substrates, thus identifying histone monoaminylation as an emerging epigenetic mark, which is highly enriched in cancer cells and possesses significant regulatory functions of gene transcription. In this review, we will summarize recent advances in TGM2-mediated histone monoaminylation as well as its role in cancer and discuss the key research methodologies to better understand this unique epigenetic mark, thereby shedding light on the therapeutic potential of TGM2 as a druggable target in cancer treatment.

## Introduction

**Figure 1:**
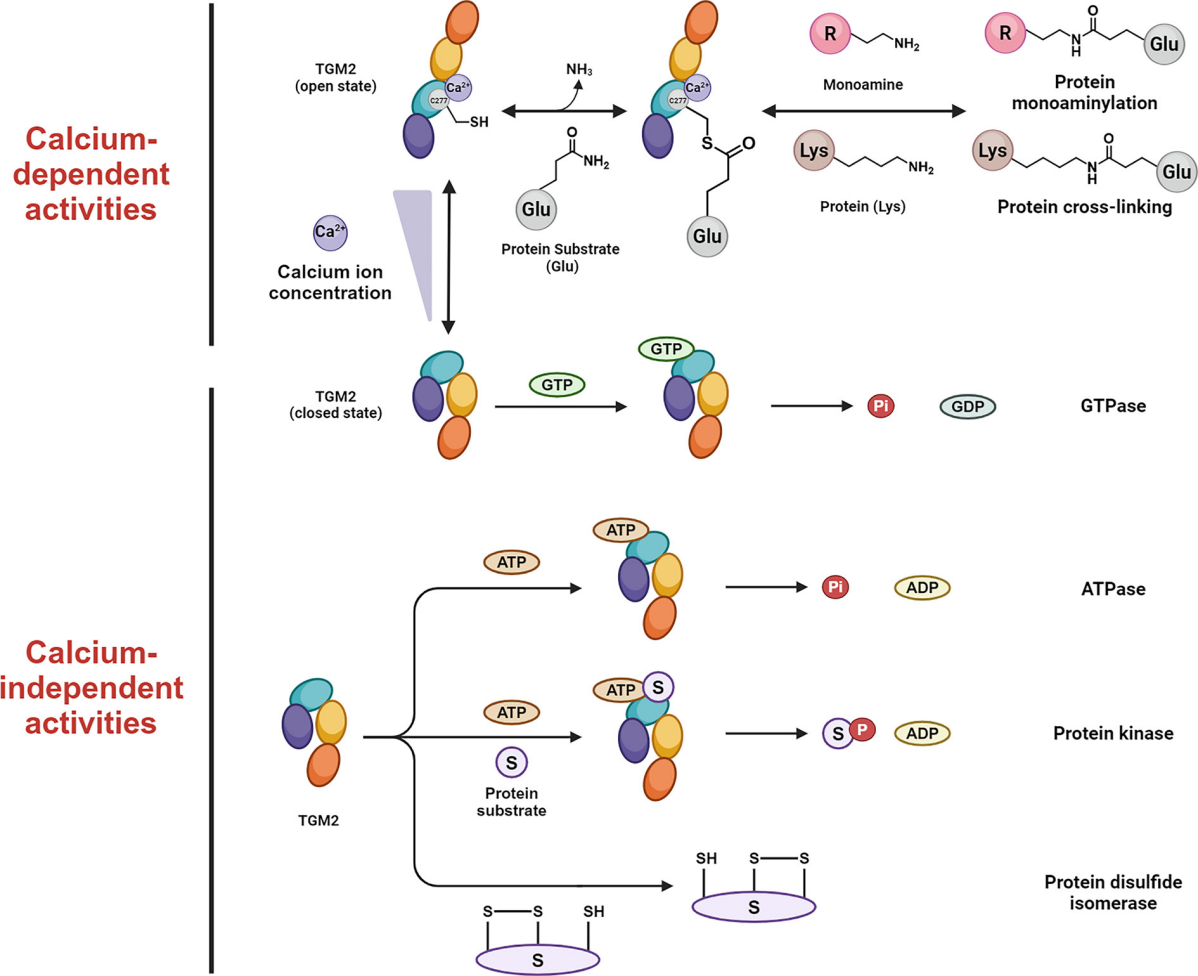
Representative activities of TGM2 TGM2 is a multifunctional protein, of which the activities can be classified to calcium dependent (such as transaminase and deaminase) and independent ones (such as GTPase, ATPase, protein kinase, and disulfide isomerase).

Transglutaminase 2 (TGM2) belongs to the transglutaminase family, which is ubiquitously expressed in diverse cell types and involved in a number of key cellular processes, including cell growth, proliferation, differentiation, migration, and apoptosis [1–3]. The multifunctional nature of TGM2 is attributed to its diverse enzymatic activities, such as transglutaminase, isopeptidase, GTPase, ATPase, protein kinase, protein disulfide isomerase (PDI), etc. (Figure 1) [4]. TGM2 was initially characterized to catalyze transamidation reactions, which are Ca^2+^-dependent and involve the incorporation of primary amine-containing molecules into substrate proteins [5–8]. Specifically, under high Ca^2+^ concentrations, TGM2 undergoes the conformational change from a closed state to an open form. Its catalytic cysteine residue (C277) attacks the γ-carboxamide group of glutamine residues within the corresponding substrate proteins to form a thioester-complex intermediate and release ammonia as a side product (Figure 1). In brief, TGM2 serves as an activation group for the protein substrates by forming reactive thioester, which can be attacked by diverse nucleophiles (e.g., amines, alcohols, and water) to result in amide, oxyester, or carboxylic acid products [8–10]. Notably, this transamidation mechanism enables the post-translational modification (PTM) and crosslinking functions of TGM2 [7,8,11,12]. The diverse PTM products formed by TGM2 are attributed to the various nucleophiles in cellular microenvironment, for example, amines for monoaminylation, alcohols for esterification, water for amide hydrolysis, and lysine for crosslinking. Intriguingly, due to its enzyme promiscuity, TGM2 has another name, ‘meat glue’, which is used for crosslinking two pieces of meat by conjugating the glutamine and lysine residues within different proteins.

While the aforementioned transamidation activity is dependent upon the activation of TGM2 by Ca^2+^, in its closed state, TGM2 can bind to GTP and function as a GTPase through a Ca^2+^-independent mechanism (Figure 1) [10,13–16]. Moreover, TGM2 has been identified to be the α-subunit of G_h_ protein complex and acts as a G-protein via α1B/α1D adrenergic receptors in cellular signaling transduction [17,18]. TGM2 also binds to ATP in a similar manner and acts as an ATPase (Figure 1) [14,19]. Notably, the GTPase activity of TGM2 inhibits its transamidase activity, while the ATPase activity does not [20]. Furthermore, TGM2 possesses kinase activity and was shown to be capable of phosphorylating insulin-like growth factor-binding protein-3 (IGFBP-3), p53, AKT, etc. [21–23] This enzymatic activity of TGM2 highly contributes to its oncogenic potential, as it is often involved in the important pathways of cell proliferation that are dysregulated in multiple types of cancer. In addition, TGM2 can function as a protein disulfide isomerase (PDI) through a separate catalytic domain [24], which was speculated to contribute to the formation of disulfide bonds in several mitochondrial respiratory complexes (Figure 1) [25]. More evidently, TGM2 interacts with and polymerizes the ADP/ATP transporter, adenine nucleotide translocator 1 (ANT1), within mitochondria and thereby affects the induction of mitochondrial pathways of apoptosis [26].

Due to its complicated enzymatic and non-enzymatic (structural) activities described above, TGM2 has been reported to play a significant role in cancer development and therapy [2,3,27,28]. Recently, histone H3 has been identified as a key substrate of TGM2, and its fifth amino acid residue, glutamine (H3Q5), was reported to undergo distinct types of monoaminylation (e.g., serotonylation, dopaminylation, and histaminylation) catalyzed by TGM2 [12]. As an emerging epigenetic hallmark, H3Q5 monoaminylation has been shown to perform important roles in regulating gene transcription, thereby impacting disease progressions. In this review, we will mainly focus on the role of TGM2 in cancer, specifically regarding its emerging regulatory function in histone monoaminylation and epigenetics. Finally, we will summarize the key technical approaches for a better understanding of TGM2-mediated histone monoaminylation and its role in cancer biology. Overall, this review will give a systematic summary of the recent research advances regarding an emerging epigenetic mark, H3Q5 monoaminylation, and the role it plays in carcinogenesis, cancer diagnosis, and therapeutics.

## Complex regulatory roles of TGM2 in cancer

TGM2 has been identified as an important factor in variant types of cancer (such as gastric cancer, ovarian cancer, colorectal cancer, breast cancer, etc.), and importantly its elevated expression level in tumor tissues is commonly linked to worse disease outcomes and poor survival rates (Table 1). Therefore, TGM2 represents not only a biomarker for cancer prognosis, but also a therapeutic target with significant clinical implications [2,3,27,28].

**Table 1:** Representative examples of the TGM2 regulatory roles in variant cancer types

Cancer type	Mechanism of action	Downstream effects
**Gastric cancer**	Inhibit TRIM21-mediated ubiquitination and degradation of STAT1 [34]	Promote cell proliferation and migration, cancer malignancy, and poorer disease prognosis [34]
**Gastric cancer**	Activate IL-1β and recruit tumor-associated macrophages, activates NF-κB [35]	Regulate tumor microenvironment and promote tumor-enhancing inflammation [35]
**Ovarian cancer**	Phosphorylate and activate ILK and GSK3α/β [36]	Enhance cancer cells adhesion to ECM and promote survival and migration [36]
**Breast cancer**	Activate the MEK/ERK/LDH pathway [42]	Increase glycolysis and promote cancer cell proliferation [42,43]
**Colorectal cancer**	Inhibit p53 and caspase-3-driven apoptosis [21,29]	Prevent cell apoptosis and promote tumorigenicity [21,29]
**T-cell lymphoma**	Activate the IL-6/JAK/STAT3 pathway [38]	Enhance the proliferation of lymphoma cells [38]
**Ependymoma**	Lead to the overexpression of ETV5	Regulate tumorigenesis [44]

**Figure 2:**
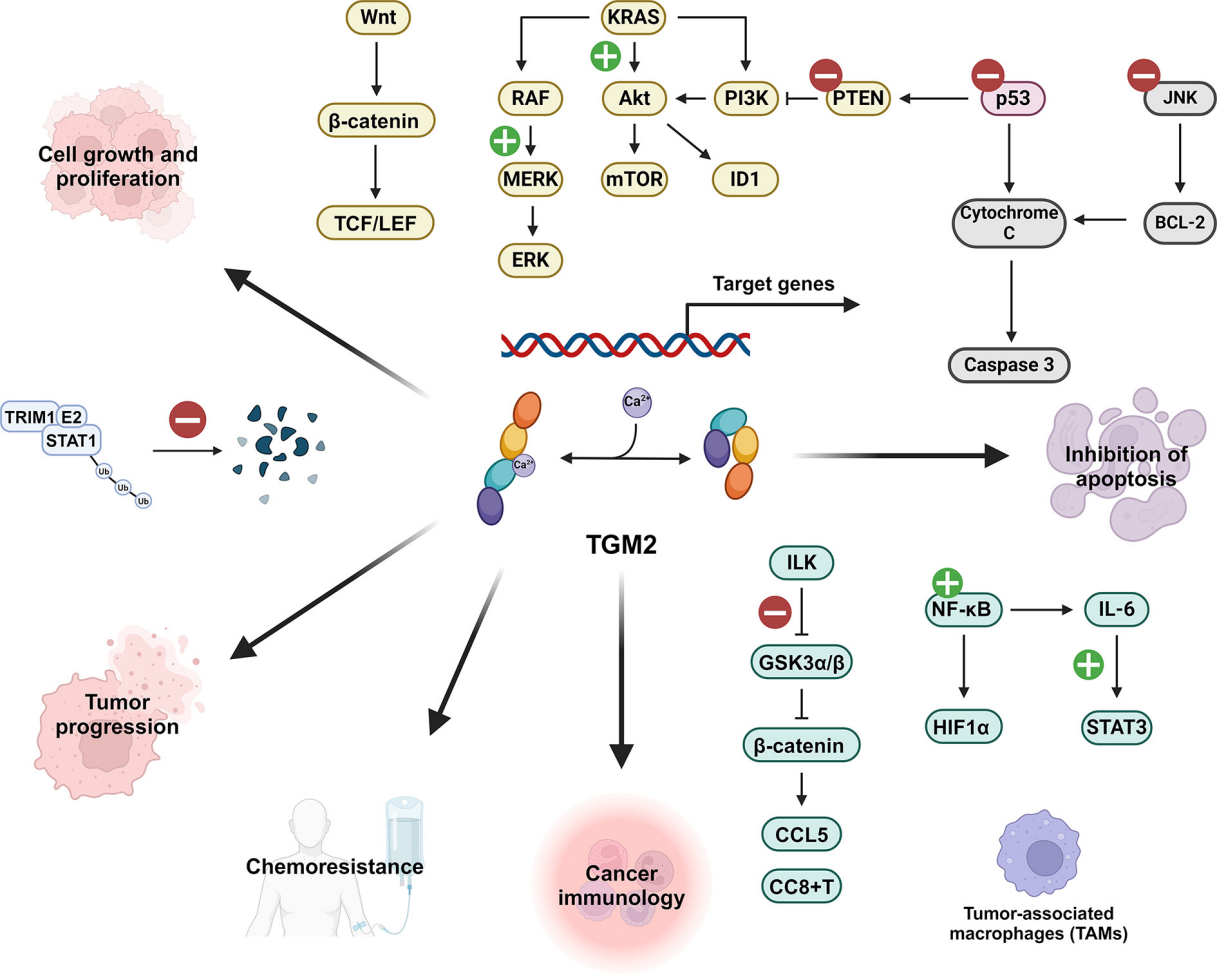
The role TGM2 plays in cancer TGM2 plays a significant but complex role in cancer progression and treatment, which is involved in a number of key signaling pathways.

Previous studies have shown that TGM2 overexpression can promote cancer cell growth *via* several cell proliferation pathways and the inhibition of apoptosis (Figure 2). TGM2 directly binds to and inhibits the tumor suppressor p53, thus hindering caspase-3-mediated apoptosis and inducing tumor escape in colorectal cancer [21,29]. TGM2 also inhibits the JNK/BCL-2 pathway, which alleviates apoptosis [30]. TGM2 interacts with multiple factors in the KRAS pathway and is associated with the aggregation of *KRAS* mutant-induced cancer development [31]. Additionally, TGM2 can up-regulate the Wnt/β-catenin pathway to inhibit cell apoptosis and promote tumorigenesis [32]. In general, the overexpression of TGM2 is linked to the up-regulation of several stemness markers, including *CD133, SOX2*, and β*-catenin*, thereby motivating cancer stem cell self-renewal pathways [33].

Moreover, TGM2 plays a constructive role in cancer immunology (Figure 2). For example, in gastric cancer, through a GTPase manner, TGM2 inhibits tripartite motif-containing protein 21 (TRIM21)-mediated ubiquitination and degradation of signal transducer and activator of transcription 1 (STAT1), and thus enhances its stability and oncogenic actions [34]. TGM2 is also associated with the recruitment of tumor-associated macrophages, which exaggerates inflammation [35]. In ovarian cancer, TGM2 interacts and activates the integrin-linked kinase (ILK) through phosphorylation at ILK Ser246, which subsequently activates β-catenin through the phosphorylation of glycogen synthase kinase-3α/β (GSK3α/β) [36]. It also activates the NF-κB pathways in ovarian cancer cells and prevents cisplatin-induced apoptosis [37]. In T-cell lymphoma cells, TGM2 up-regulates the IL-6/JAK/STAT3 pathway, which proliferates the growth of T-cell lymphoma cells [38].

Previous research has also shown that TGM2 is closely related to cancer treatment efficacy (Figure 2). For example, elevation of TGM2 has been found to account for the resistance to multiple cancer therapies, while down-regulation of TGM2 alleviates chemoresistance and enhances the therapeutic effects [39,40]. Specifically, TGM2 has significant impacts on doxorubicin resistance in cancer treatment [40] and mediates the resistance to neratinib in metastatic breast cancer by up-regulating IL-6 and activating the NF-κB signaling pathways [41].

Even though the correlation between TGM2 and cancer is clear, the detailed regulatory mechanisms are still poorly understood. As the overexpression of TGM2 in cancer cells is usually linked to transcription level changes of cancer-related genes, TGM2-mediated histone monoaminylation may serve as a key upstream regulation mechanism to manipulate gene transcription in an epigenetic fashion. A better understanding of the biochemical and genetic basis of TGM2-mediated histone monoaminylation will facilitate the development of new anti-cancer strategies in the future.

## TGM2-mediated histone monoaminylation

**Figure 3:**
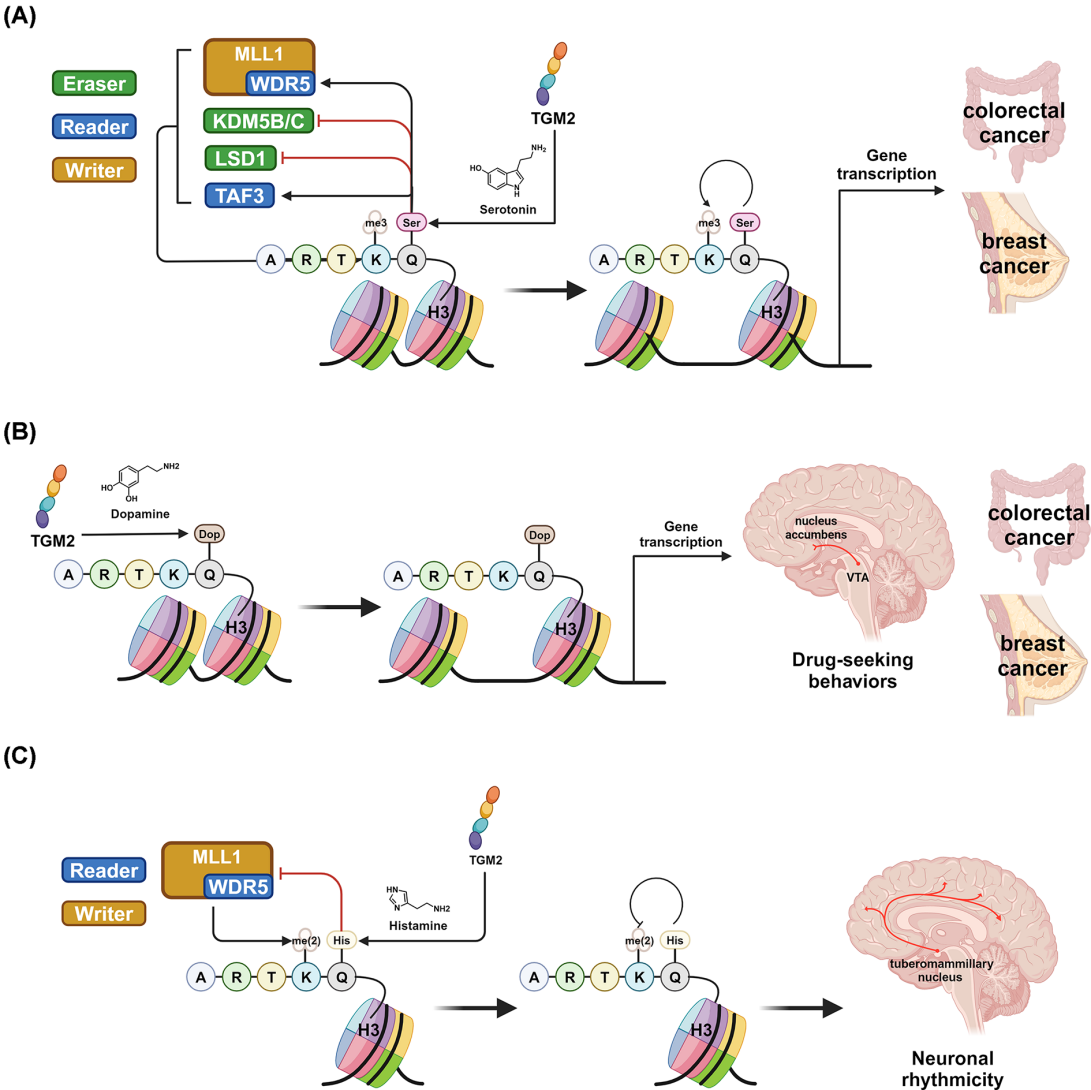
Overview of the epigenetic regulations caused by histone monoaminylation TGM2-mediated histone monoaminylations, including H3Q5ser (**A**), H3Q5dop (**B**), and H3Qhis (**C**), function as an emerging epigenetic mark and exhibit significant impacts on gene transcription in both health and disease states, especially neurological disorders and cancer.

Histone proteins (i.e., H1, H2A, H2B, H3, and H4) package and interact with DNA to form the dynamic chromatin structure in eukaryotic cells and regulate gene expression through transcriptional effects without influencing the DNA sequences. Histone PTMs (also referred to as the ‘histone code’) play critical roles in this epigenetic regulation process, as they contribute to the relaxation or condensation of cellular chromatin and interact with variant mediators (e.g., transcription factors). Recently, glutamine residues on core histones have been found to be the substrate of TGM2, which could undergo the transamidation reaction to incorporate a monoamine (such as serotonin, dopamine, or histamine) as the PTM donor and form an isopeptide bond (Figure 3) [45]. This TGM2-mediated enzymatic process was characterized as a new category of histone PTMs, i.e., histone monoaminylation. There are a number of monoamines that can stoichiometrily bind to TGM2 *in vitro* and thus function as the amine donor for its transamidation activity [6]. Our previous biomedical investigations indicated that TGM2-mediated histone monoaminylation is a cellular microenvironment-driven PTM, as diverse monoamine metabolites can act as nucleophiles to attack the thioester complex of TGM2 and H3 [12]. Currently, the most-widely-studied histone monoaminylation that occurs naturally *in vivo* is histone H3 serotonylation on its Q5 residue (H3Q5ser) [46]. Other reported examples of histone monoaminylation include H3Q5 dopaminylation (H3Q5dop) and histaminylation (H3Q5his) [12]. As serotonin, dopamine, and histamine are all important signaling metabolites that are enriched in tumor microenvironment, TGM2-mediated histone monoaminylation is supposed to serve as a biomarker accumulated in cancer cells and extensively regulate cancer-related genes in an epigenetic manner.

### H3Q5 serotonylation (H3Q5ser)

The first discovered and most-widely-studied histone monoaminylation is H3Q5 serotonylation (H3Q5ser) [46]. H3Q5ser was identified using a chemical probe (i.e., 5-propargylated tryptamine) and biorthogonal labeling approach, where H3 was detected as a substrate of TGM2 within the serotonylated nuclear protein component and H3Q5 was found to be the dominant modification site [46]. H3Q5ser is generally known as a permissive epigenetic marker that promotes euchromatin and transcriptional activation (Figure 3A).

Due to its close proximity to the well-studied histone PTM site H3K4, the cross-talk between H3Q5ser and H3K4me3 has been further characterized [46–50]. While the serotonylation on H3Q5 is not affected by the presence of H3K4me3, the dual-modification, H3K4me3Q5ser, rather than H3Q5ser alone, is most enriched *in vivo* in tissues that have high local concentrations of serotonin and correlated with transcriptional activation, especially during cellular differentiation [46]. The presence of H3Q5ser enriches H3K4me3 level and potentiates the genetic activating consequences of H3K4me3 through variant approaches [50]. For example, H3Q5ser interacts with transcription factor II D (TFIID), of which the subunit TATA-box binding protein associated factor 3 (TAF3) was reported to be a ‘reader’ for H3K4me3, and enhances its binding to H3K4me3 (Figure 3A) [46]. Specifically, the addition of H3Q5ser next to H3K4me3 makes it more favorable bound to the PHD finger of TAF3 [48]. H3Q5ser also stabilizes the H3K4me3 mark by inhibiting H3K4me3 ‘erasers’, such as KDM5B/C and LSD1, and blocking its demethylation (Figure 3A) [48]. Additionally, the currently reported ‘reader’ of H3Q5ser, WD repeat-containing protein 5 (WDR5), which is a component of the mixed lineage leukemia (MLL) complex, interacts with H3K4me3 and is associated with its deposition (Figure 3A) [49,51–53].

H3K4me3 is highly implicative in the progression of variant types of cancer including breast cancer, colorectal cancer, and pancreatic cancer, while its functional impacts tend to be complicated and tissue-specific [47,52,54,55]. The cross-talk between H3Q5ser and H3K4me3 even furthers this complicacy. Current knowledge on the mechanisms of H3Q5ser remains insufficient to understand its direct functions and specific roles in cancer. Using the novel chemical biology tools and bioorthogonal labeling approaches, we demonstrated that H3Q5ser was enriched in breast and colon cancer cells [50]. Notably, H3Q5ser exerts transcriptional manipulation in cancer cells not only through the cross-talk with H3K4me3, but also directly decompresses chromatin structures through the steric effect [48]. More recently, H3Q5ser was found to regulate the tumorigenesis of ependymoma, in which the blockage of H3Q5ser inhibits tumor formation [44]. The ETS variant transcription factor 5 (ETV5) is one of the key downstream effectors of H3Q5ser in ependymoma and has important roles in both neuronal development and chromatin regulation [44].

### H3Q5 dopaminylation (H3Q5dop)

Dopamine is a well-known neurotransmitter in the central nervous system and plays vital roles in reward activity, learning, and emotions [56,57]. Similar to H3Q5ser, H3Q5 dopaminylation (H3Q5dop) is catalyzed by TGM2, where dopamine serves as the nucleophile and PTM donor (Figure 3B) [58]. H3Q5dop was found to regulate drug addiction activities through transcriptional influences, regardless of H3K4me3. The increased level of H3Q5dop in ventral tegmental area (VTA), where 65% of dopaminergic neurons locate, was found in male rats during the withdrawal periods after cocaine and heroin administration, which accounted for the drug-induced transcriptional plasticity regulating neuronal signaling and resulted in drug-seeking behaviors [58,59]. Notably, reducing the H3Q5dop level was able to reverse this process and alleviate drug-induced changes in gene expression. H3Q5dop also accumulates in nucleus accumbens (NAc), downstream of neuronal signaling from VTA, and as well regulates cocaine-induced gene expression alterations and related behaviors [60].

Dopamine has also been identified as an important phenylalanine-derived metabolite in tumor microenvironment, which was found to either promote or inhibit tumor cell growth through different receptor-mediated signaling pathways (e.g., G protein-coupled dopamine D2 receptor (DRD2)) [61]. The characterization of the emerging epigenetic mark, H3Q5 dopaminylation, provides a novel mode of action (MOA) caused by dopamine in cancer biology. Notably, due to the enzyme promiscuity of TGM2, dopaminylation occurs not only on histones, but glutamine residues of diverse cellular proteins. Utilizing a bicyclononyne (BCN)-based chemical probe, we recently labeled and enriched dopaminylated proteins in a biorthogonal manner [62]. Therefore, a dopaminylation proteome of over 400 dopaminylated proteins was identified from cancer cells. These dopaminylated proteins mostly localize in the nucleus (72%) and affect gene transcription. As expected, H3Q5dop was also found to accumulate in colorectal and breast cancer cells (Figure 3B) [62], which could be attributed to the high expression level of TGM2 in these cells, further highlighting its close association with cancer progression.

### H3Q5 histaminylation (H3Q5his)

Histamine is an active biogenic amine that mainly functions as an immune regulator of allergic reactions [63]. It is also known as a neurotransmitter that signals through G protein-coupled histamine receptors [64]. TGM2-meditated histaminylation on protein glutamine residues has been identified and shown to play important roles in both inflammatory and neuronal signaling pathways [65,66]. We recently revealed H3Q5 histaminylation (H3Q5his) as another example of histone monoaminylation in cells and brain tissues [12]. H3Q5his occurs endogenously in cells expressing TGM2 and disappears upon TGM2 functional mutation (C277A). Interestingly, H3Q5his is highly dynamic, and both ‘written’ and ‘erased’ by TGM2. Moreover, the histaminylated H3 could also function as a substrate of TGM2 to be further ‘re-wrote’ and converted to other monoaminylations (such as dopaminylation and serotonylation) in the presence of corresponding amine donors [12]. Importantly, H3Q5his is highly enriched in the posterior hypothalamic tuberomammillary nucleus (TMN), which contains mostly histaminergic neurons, displays rhythmic expression patterns, and associates with the expression pattern of several circadian genes and related behaviors. Like H3Q5ser, H3Q5his can also co-occur with H3K4 methylation, while its rhythmicity feature is linked to H3K4me2 rather than H3K4me3 (Figure 3C). Conversely, H3Q5his antagonizes H3K4me2 level by perturbing MLL1 methylation activity on H3K4 through the repulsion against its subunit, WDR5 (Figure 3C) [12]. Even though the pathophysiological relevance of H3Q5his in cancer has not yet been reported, its novel MOA in epigenetic regulations (such as the cross-talk with H3K4me2) is believed to have significant impacts on cancer development and therapeutics.

## Novel methodology to study histone monoaminylation in cancer

Our recent discoveries regarding the high accumulation of TGM2-mediated histone monoaminylation in cancer cells and tumor tissues have shed light on its novel regulatory functions in cancer as an emerging epigenetic hallmark. However, due to the lack of efficient tools (e.g*.*, site-specific and pan-specific antibodies) and methodologies, the detailed pathophysiological roles of histone monoaminylation in cancer development and treatment still remain elusive. The development of novel tools and methodologies to study TGM2-mediated histone monoaminylation in cancer is urgently needed.

### Manipulating the transamidation activity of TGM2

The conventional and currently most accessible strategy for studying histone monoaminylation is to manipulate the transamidation activity of TGM2 by using medicinal chemistry or molecular biology approaches. For instance, ectopic overexpression of TGM2 enhances its transamidation activity and histone monoaminylation levels. HEK293T cell line is commonly used as a negative control, as it does not express endogenous TGM2 [12]. Notably, the catalytically inactive mutation of TGM2, C277A, can completely abolish its transamidation activity and therefore is used as a negative control for TGM2-overexpression experiments. On the contrary, short hairpin RNA (shRNA) targeting *tgm2* is commonly used for gene knockdown of TGM2.

**Figure 4:**
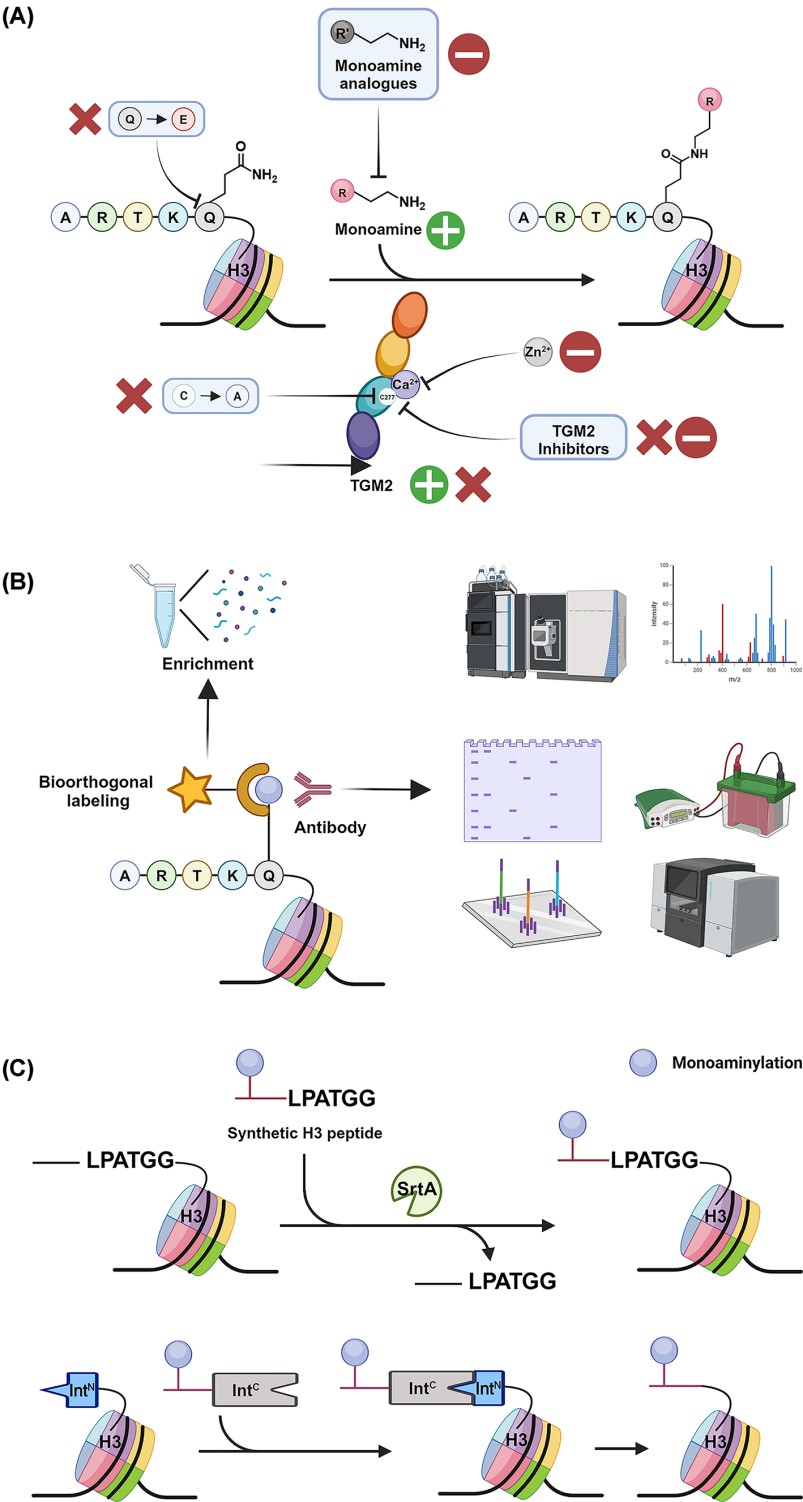
Overview of the methodologies for studying histone monoaminylation Novel medicinal chemistry (**A**), chemical biology (**B**), and synthetic biology (**C**) approaches for future investigations regarding TGM2-mediated histone monoaminylation and its role in cancer.

Moreover, TGM2 loss-of-function approaches involve the delivery of TGM2 inhibitors (Figure 4A). Well-established TGM2 inhibitors function through similar routes by reversibly or irreversibly binding to its key regulatory site or substrates. Competitive TGM2 inhibitors that mimic amine donors include cystamine, spermidine, putrescine, *etc* [67]. As described above, the binding of GTP (and its analogues) can also exclude its transamidase activity. The metal ion Zn^2+^ competes with Ca^2+^ in binding TGM2, thereby attenuating its Ca^2+^-dependent transamidation activity [67]. ZM39923, ZM449829, tyrphostin 37, and vitamin K3 were also known to inhibit TGM2 with considerable IC_50_ through a thiol-dependent mechanism of action on its key cysteine residue [68]. Besides, there are irreversible TGM2 inhibitors that covalently target the active cysteine site, C277 [67]. Overall, these inhibitors have been widely used for *in vitro* and *in vivo* studies of TGM2-mediated protein monoaminylation (Figure 4A).

Other interventions for TGM2 transamidation activity involve the control of monoamine donor levels and its substrates. Specifically, treating the samples with exogenous monoamines (e.g., serotonin, dopamine, and histamine) enhances the corresponding monoaminylation levels [12,46,58]. Additionally, the modification of H3Q5 to inactive substrates prevents the deposition of histone monoaminylation. For example, H3Q5E mutation has been commonly used as a negative control, which in fact is the deamidation product of H3 catalyzed by TGM2 and cannot undergo further monoaminylation as a ‘dead-end product’ (Figure 4A) [12]. This approach was employed in a recent study discovering the regulatory role of H3Q5ser in ependymoma and the H3Q5E mutation was utilized as a ‘dominant-negative form’ of the serotonylated H3 [44].

To note, given the complicacy and diversity of TGM2 activities and substrates, manipulation strategies described above typically tend to raise unspecific side consequences, which request tight controls and easily confound the analyses of experimental results. More direct and easily manipulable strategies are in need for better understandings of TGM2-mediated histone monoaminylation in cancer.

### Bioorthogonal labeling and enrichment of histone monoaminylation

H3Q5 monoaminylation labeling and enrichment strategies are commonly applied for immunoprecipitation (IP) and proteomic profiling experiments. The antibodies targeting H3Q5ser, H3Q5dop, and H3Q5his, along with the ones recognizing bivalent histone PTMs (i.e., H3K4me3Q5ser, H3K4me3Q5dop, and H3K4me3Q5his) have been generated and established in previous studies for pull-down assays, Western blotting, and ChIP-Seq (Figure 4B) [12,46,58]. However, there are obvious limitations to these tools, which greatly restrict their scope of applications. For example, many of these antibodies could not be utilized for *in vivo* studies and none of them can be employed for global profiling of monoaminylation proteome in cancer cells.

Recently, we have successfully developed a series of chemical probes, which are more sensitive than the corresponding antibodies, to specifically label and enrich H3Q5 monoaminylation as well as other monoaminylated proteins in cancer cells via biorthogonal chemistry (Figure 4B) [50,62,69]. These powerful chemical biology tools are applicable not only for the proteomic profiling of monoaminylated proteins in cancer cells, but also for pull-down assays to identify protein–protein and protein–DNA/RNA interactions (Figure 4B). As described above, we were able to characterize the high accumulation level of histone monoaminylation in cancer. Overall, these newly developed tools will largely facilitate the future mechanistic research of TGM2-mediated histone monoaminylation.

### Repurposing protein ligation strategies for depositing histone monoaminylation

As described above, TGM2 possesses multiple functions in cancer development and treatment. To distinguish the role of TGM2-mediated histone monoaminylation with the other TGM2 activities in cancer, novel methodologies for depositing histone monoaminylation without overexpressing TGM2 are in need. Naturally occurring and engineered enzymes are capable of ligating the synthetic peptides with tagged proteins *in vitro* and *in vivo*, thereby delivering the PTMs of interest [70]. Combining peptide synthesis and enzymatic protein ligation strategies will provide a flexible approach to edit the ‘histone code’ and engineer protein PTMs of interest. The split intein- and sortase-based methods are two examples widely used to investigate histone PTMs.

Split inteins are a powerful tool in protein chemistry that undergo spontaneous splicing reactions for *in vitro* and *in vivo* protein ligations [71]. To deliver histone H3 PTMs using split inteins, a truncated N-terminus of H3 with Int^C^ (Int^C^-H3) is first expressed and incorporated into the cellular chromatin. Thereafter, an Int^N^-fused peptide sequence containing the histone PTM(s) of interest is exogenously delivered into cells, so that it fuses with the Int^C^ tag on histone H3 to enable the rational engineering of epigenetic marks within chromatin [72,73]. This strategy is applicable for *in vitro* and *in vivo* site-specific histone manipulation, which is promising for histone monoaminylation studies (Figure 4C).

Sortase A (SrtA) is a transpeptidase discovered from *Staphylococcus aureus* [74]. It reversibly catalyzes the transpeptidation reaction by recognizing and cleaving (between the threonine and glycine residue) the ‘LPXTGG’ motif, thereby enabling the ligation between LPXTGG-tagged proteins and polyG-containing peptide/protein donors [70,74]. This mechanism of action allows for the rational engineering of both N- and C-terminal sequences of the target protein. More importantly, the ‘LPXTGG’ motif cleaved by SrtA is only one-amino-acid different from the wild-type histone H3 sequence ‘APATGG’ (aa 29-34), which enables the bioorthogonal engineering of mutant H3 containing the ‘LPATGG’ sequence (i.e., H3A29L) [75]. Combining the overexpression of H3A29L together with SrtA and delivery of synthetic H3 N-terminal peptides containing the PTM(s) of interest enables the deposition of target histone PTM(s) both *in vitro* and in live cells (Figure 4C) [75]. Interestingly, this sortase-mediated strategy has also been optimized and successfully employed for engineering histones H2B and H4 [76]. Overall, the aforementioned enzymatic protein ligation approaches can be applied for introducing histone monoaminylation into cancer cells without influencing other TGM2-involved signaling pathways, which are ideal for understanding the role of TGM2-mediated histone monoaminylation in cancer (Figure 4C).

## Summary and perspectives

Previous studies have shown that the various enzymatic and non-enzymatic activities of TGM2 widely affect the signaling transductions in cancer cells (Figures 1 and 2; Table 1). The discovery of TGM2-mediated histone monoaminylation added a new MOA of TGM2 in cancer biology, which regulates gene transcription and chromatin structure in an epigenetic manner (Figure 3). Currently reported histone monoaminylations (i.e*.*, H3Q5ser, H3Q5dop, and H3Q5his) exhibit distinct biological effects in regulating gene transcription, which are interrelated with H3K4 methylation. Therefore, current understandings about the MOA of TGM2-mediated histone monoaminylations in cancer are basically based on its cross-talk with other histone PTMs (such as H3K4me3 and H3K4me2) and indirect impacts on epigenetic regulations. The knowledge of its direct epigenetic effects in cancer cells is still lacking, for example, the *bona fide* readers for each histone monoaminylation type. Moreover, as the PTM donors for histone monoaminylation are well-known neurotransmitters, previous studies regarding TGM2-mediated histone monoaminylation are mostly in the field of neuroscience. To understand its pathophysiological relevance in cancer is a promising direction in the future.

The development of novel chemical biology and synthetic biology approaches will facilitate the research regarding TGM2-mediated histone monoaminylation in cancer (Figure 4). For example, our recent advances of chemical tool development successfully revealed that histone monoaminylation was highly enriched in tumor cells and a large number of key regulatory proteins were monoaminylated in cancer cells. These discoveries suggested that histone H3Q5 monoaminylation might serve as a biomarker for cancer early diagnosis and TGM2 should become a promising druggable target for cancer therapies in clinic. Moreover, epigenome engineering approaches (e.g*.*, split intein- and sortase-based *in vivo* histone ligation) can further the understandings of TGM2-mediated histone monoaminylation in cancer without interrupting other TGM2-related signaling pathways. Overall, TGM2-mediated histone monoaminylation is an emerging epigenetic hallmark with many unknown functions and will become an exciting research direction in cancer biology, while the development of novel tools and methodologies is needed.

## Abbreviations

ANT1: adenine nucleotide translocator 1
BCN: bicyclononyne
GSK3α/β: glycogen synthase kinase-3α/β
IGFBP-3: insulin-like growth factor-binding protein-3
MLL: mixed lineage leukemia
MOA: mode of action
SrtA: Sortase A
STAT1: signal transducer and activator of transcription 1
TGM2: transglutaminase 2
TMN: tuberomammillary nucleus
VTA: ventral tegmental area
